# CAEV Vif Hijacks ElonginB/C, CYPA and Cullin5 to Assemble the E3 Ubiquitin Ligase Complex Stepwise to Degrade oaA3Z2-Z3

**DOI:** 10.3389/fmicb.2019.00565

**Published:** 2019-03-19

**Authors:** Zhilei Zhao, Zhaolong Li, Chen Huan, Hong Wang, Xing Su, Wenyan Zhang

**Affiliations:** Institute of Virology and AIDS Research, The First Hospital of Jilin University, Changchun, China

**Keywords:** CAEV-Vif, APOBEC3, interplay mechanism, E3 ubiquitin ligase, functional domain

## Abstract

Caprine arthritis encephalitis virus (CAEV) is a lentivirus that causes multisystemic chronic disorders in sheep and goats. It encodes Vif to counteract the restriction of *Ovis aries* A3Z2-Z3 (oaA3Z2-Z3) by inducing their degradation. Nevertheless, the mechanisms underlying the interplay between CAEV Vif and OaA3Z2-Z3 have yet to be elucidated. Here, we identified the cellular factors ElonginB/C, CYPA and Cullin5 as being hijacked by CAEV Vif as well as several functional domains of CAEV Vif required for degrading oaA3Z2-Z3. Moreover, we determined that CAEV Vif assembled E3 ubiquitin ligase stepwise via its SLE motif (170SLE172) to recruit ElonginB/C, the P21 site and the zinc finger motif (C132-C134-C154-C157) to recruit CYPA, as well as the hydrophobic domain (141IR142) to recruit Cullin5. And this CAEV Vif-mediated E3 ligase triggers the proteasomal degradation of oaA3Z2-Z3, which directly bind CAEV Vif through residues Y39 and L44. In particular, CYPA played an essential role in the process to regulate ligase assembly, which was analogous to CBF-β, the essential regulator for HIV-1 and SIV-mediated E3 ligase, indicating that there is a modular conservation and lineage-specific preference for cellular partners required by Vifs from different subgroups of lentiviruses. Taken together, these findings provide important insights regarding the CAEV Vif function and deepen our understanding of the arms race between the lentiviruses and their hosts.

## Introduction

During their long-term co-evolution, lentiviruses and their hosts have developed a series of infective-defensive interplays (Harris et al., 2012; Harris and Anderson, 2016). The APOBEC3 (apolipoprotein B mRNA-editing catalytic polypeptide-like 3, A3) proteins are mammalian-specific host restrictive factors that are packaged into virions and catalyze the deamination of cytosine to uracil during reverse transcription (Harris and Liddament, 2004; Harris and Dudley, 2015; Harris and Anderson, 2016). Accordingly, nearly all lentiviruses, except equine infectious anemia virus (EIAV) (Kawakami et al., 1987) and the extinct rabbit endogenous lentivirus RELIK (Katzourakis et al., 2007), encode the Vif protein to neutralize the antiviral activity of APOBEC3 by recruiting host co-factors to trigger the proteasomal degradation of APOBEC3 proteins (Conticello et al., 2003; Gaddis et al., 2004; Zhang et al., 2008, 2016; Larue et al., 2010; Wang et al., 2011; Zhang J. et al., 2014; Zhang W. et al., 2014). Despite their conserved function, the cellular factors required by various lentiviruses are diverse. For example, the HIV-1 Vif hijacks Cullin5 (CUL5), ELOB/C and CBF-β to neutralize the antiviral activity of human A3 proteins (Jager et al., 2011; Zhang et al., 2011), while Cullin 2 (CUL2), ELOB/C, and RBX1, but not CUL5 or CBF-β, are used by bovine immunodeficiency virus (BIV) and Jembrana disease virus (JDV) Vif to form a CRL2 E3 ubiquitin ligase to degrade the restrictive bovine A3 proteins (Zhang W. et al., 2014; Su et al., 2018).

Caprine arthritis encephalitis virus (CAEV) is a pathogen that causes multisystemic chronic disorders in sheep and goats. Together with Maedi-Visna virus (MVV), CAEV belongs to the small ruminant lentivirus (SRLV) subgroup (Minardi da Cruz et al., 2013; Ramirez et al., 2013). Due to the negative impact of these viruses on animal production and welfare, the mechanism of and strategy against SRLV infection needs to be further explored. Recently, the way in which MVV counteracts with the host has been investigated in a growing number of studies (Jonsson and Andresdottir, 2013). The Vif protein is crucial for MVV replication both *in vivo* and *in vitro* (Kristbjornsdottir et al., 2004). *Ovis aries* (sheep) encodes at least four A3 proteins, A3Z1, A3Z2, A3Z3 and A3Z2-Z3 (Nakano et al., 2017). According to phylogenetic and subcellular distribution analyses, sheep A3Z1 was found to correspond to human A3A, sheep A3Z3 corresponded to human A3H and sheep A3Z2-Z3 corresponded to human A3F and A3G (Jonsson and Andresdottir, 2013), and they display significant anti-HIV-1 activity. MVV Vif has been demonstrated to overcome the restriction of *Ovis aries* A3Z2-Z3 (oaA3Z2-Z3) (Larue et al., 2010) by recruiting CUL5 to facilitate its degradation in a proteasome-dependent manner (Zhang J. et al., 2014). In addition, core-binding factor β (CBF-β), which was a critical regulator of HIV-1 Vif function, has no effect on MVV Vif activity (Ai et al., 2014; Kane et al., 2015). Instead, a novel cofactor, cyclophilin A (CYPA), was required by MVV to form a stable CRL complex (Kane et al., 2015; Yoshikawa et al., 2016). CYPA was found to bind directly to residues P21 and P24 of MVV Vif and serve an analogous role as CBF-(β in E3 ligase formation by maintaining the stability of the MVV Vif-ELOB/C complex (Kane et al., 2015). Compared with the comprehensive interpretation of MVV infection and restriction, the host-virus interplay of CAEV Vif has rarely been studied, except that CYPA was also hijacked by CAEV Vif to degrade *Ovis aries* A3 (Kane et al., 2015; Yoshikawa et al., 2016). Additional host factors employed by CAEV Vif as well as its domain distribution need to be identified.

In the present study, we investigated the cellular requirement and functional domain of CAEV Vif. The cellular factors ElonginB/C, CYPA and Cullin5, but not CUL2 or CBFβ, were hijacked by CAEV Vif. Following site-directed mutagenesis and co-immunoprecipitation, we observed that the E3 ubiquitin ligase complex induced by CAEV Vif was assembled in a stepwise fashion. By binding ELOB/C at the SLE motif in the BC box (170SLE172), CAEV Vif-ELOB/C forms a substrate receptor; then, cellular factor CYPA played a similar role as CBFβ in regulating the ligase assembly by associating with CAEV Vif-ELOB/C on residue P21 as well as the zinc finger motif (C132-C134-C154-C157) to facilitate CUL5 binding at the hydrophobic domain (141IR142). In particular, CYPA played an essential role in this assembly process that silenced the endogenous CYPA or CYPA-binding site mutation, significantly reducing the CAEV Vif-CUL5 association. Moreover, residues Y39 and L44 of CAEV Vif contribute to its interaction with oaA3Z2-Z3. Taken together, these findings will deepen our understanding of CAEV infections and may be beneficial for future pharmaceutical design (Salter et al., 2014).

## Materials and Methods

### Plasmid Construction

The HIV-1 Vif-deficient molecular clone NL4-3ΔVif was obtained from the AIDS Research and Reference Reagents Program, Division of AIDS, National Institute of Allergy and Infectious Diseases (NIAID), National Institutes of Health (NIH). The VR1012 vector was a gift from Vical (San Diego, CA, United States). The full-length CAEV Vif was synthesized by Shanghai Generay Biotech Co. and subcloned into the VR1012 vector with a HA tag at the C-terminus. The CAEV Vif derived mutants were constructed by PCR-based mutagenesis assay. Expression vectors for HIV Vif-HA, UBE2F-Flag (C116S) and UBE2M-Flag (C111S) have been described previously (Yu et al., 2003; Zhang W. et al., 2014). The primers for plasmid construction are listed in Table 1, and all constructs used in the present study were verified by sequencing.

**Table 1:** Primers used for plasmid construction in this study.

Construction	Primer direction	Sequence (5′-3′)
CAEV Vif SLE-AAA	Forward	GCATACTAGAACAAAAGCTGCGGCTAGACTAGTATTGCTG
	Reverse	GCTTTTGTTCTAGTATGCTTTACTACTTC
CAEV Vif-Y39A	Forward	AAGAGAGCTCATGGGCCATAACAGTAAGACTAC
	Reverse	GCCCATGAGCTCTCTTTATTGATGC
CAEV Vif-I40A	Forward	GAGAGCTCATGGTACGCAACAGTAAGACTACAAC
	Reverse	GCGTACCATGAGCTCTCTTTATTG
CAEV Vif-T41A	Forward	GAGAGCTCATGGTACATAGCAGTAAGACTACAACAGATG
	Reverse	GCTATGTACCATGAGCTCTCTTTATTGATGC
CAEV Vif-V42A	Forward	GAGCTCATGGTACATAACAGCAAGACTACAACAGATGATG
	Reverse	GCTGTTATGTACCATGAGCTCTCTTTATTGATGC
CAEV Vif-R43A	Forward	CATGGTACATAACAGTAGCACTACAACAGATGATGTGG
	Reverse	GCTACTGTTATGTACCATGAGCTCTC
CAEV Vif-L44A	Forward	GGTACATAACAGTAAGAGCACAACAGATGATGTGGAAC
	Reverse	GCTCTTACTGTTATGTACCATGAGCTCTC
CAEV-Vif-P18A	Forward	ATAGGGAACAGGGAGCAGAATTACCATTAGC
	Reverse	CTCCCTGTTCCCTATTCCTTCTCC
CAEV-Vif-P21A	Forward	CAGGGACCAGAATTAGCATTAGCATTGTGG
	Reverse	CTAATTCTGGTCCCTGTTCCCTATT
CAEV Vif-H122L	Forward	AAAAGGTAGACAGGCTCTTCTGGTGG
	Reverse	AGCCTGTCTACCTTTTTGTAATCTCC
CAEV Vif-C132S	Forward	GGCGGATAATGTTGTCTTCATGCAGG
	Reverse	GACAACATTATCCGCCATGCCCACCA
CAEV Vif-C134S	Forward	TAATGTTGTGTTCATCCAGGAAAGAA
	Reverse	GATGAACACAACATTATCCGCCATGC
CAEV Vif-H149L	Forward	TTCTGAGAGGAAGGCTTAGATGGGAT
	Reverse	AGCCTTCCTCTCAGAAATTCTCTTAT
CAEV Vif-C154S	Forward	ATAGATGGGACTTGTCAAAATCCTGTG
	Reverse	TGACAAGTCCCATCTATGCCTTCCTCT
CAEV Vif-C157S	Forward	ACTTGTGTAAATCCTCTGCTCAGGGA
	Reverse	GAGGATTTACACAAGTCCCATCTATG
CAEV Vif-H165L	Forward	GAGAAGTAGTAAAGCTTACTAGAACA
	Reverse	AGCTTTACTACTTCTCCTTGAGCACA
oaA3Z2-Z3-Flag	Forward	GCGTCGAC ATGCCCTGGATCAGCGAC
	Reverse	TAAAGCGGCCGCTCACTTGTCATCGTCGTCCTTGTAGTC AGTCGGCGCCGTCAGGATC
shCYPA	Forward	CCGGAACTTCATCCTAAAGCATACGCTCGAGCGTATGCTTTAGGATGAAGTTTTTTTG
	Reverse	AATTCAAAAAAACTTCATCCTAAAGCATACGCTCGAGCGTATGCTTTAGGATGAAGTT

### Cell Culture and Transfection

Human embryonic kidney 293T (HEK293T) (catalog no. CRL-11268) cells were obtained from American Type Culture Collection (ATCC; Manassas, VA, United States) and cultured in Dulbecco’s modified Eagle’s medium (HyClone, Logan, UT, United States) with 10% fetal bovine serum (FBS; PAN-Biotech, Germany) and penicillin-streptomycin.

HEK293T cells were transfected using Lipofectamine 2000 (Invitrogen) according to the manufacturer’s protocol. For degradation experiments, HEK293T cells seeded in 12-well plates were transfected with 300 ng of CAEV Vif or VR1012 and 100 ng of oaA3Z2-Z3. For viral infectivity assays, HEK293T cells in 12-well plates were transfected with 500 ng of NL4-3ΔVif, 300 ng of CAEV Vif and 100 ng of oaA3Z2-Z3. For immunoprecipitation assays, HEK293T cells in 6 cm-dishes were transfected with 5 μg of VR1012 or Vif.

### Inhibitor Treatments

To explore whether the CAEV Vif induced oaA3Z2-Z3 degradation via proteasomal pathway, MG132 (Catalog No. C2211; Sigma) inhibition assay was performed. At 36 h post transfection with indicated plasmids, HEK293T cells were treated with the 10 μM MG132proteasome inhibitor or dimethyl sulfoxide (DMSO) as a negative control for another 12 h. The cells were then harvested and analyzed by immunoblotting.

To investigate whether zinc coordination is critical for the CAEV Vif mediated-degradation of oaA3Z2-Z3, HEK293T cells were treated with 2.5 μM zinc chelator N, N, N′-tetrakis-(2′-pyridylmethyl) ethylenediamine (TPEN, catalog no. P4413; Sigma) or DMSO as a negative control at 24 h post transfection. At 48 h after transfection, the cells were harvested and detected by immunoblotting.

### Virus Purification and Viral Infectivity Assay

At 48 h after transfection with indicated plasmids, the cell culture medium was collected and cleared by centrifugation at 10,000 × *g* for 5 min in a Sorvall RT 6000B centrifuge as well as filtration through a 0.22-μm-pore-size membrane (Millipore) to remove the cell debris. Virus particles were then concentrated by ultracentrifugation through a 25% sucrose cushion at 100,000 × *g* for 2 h at 4°C in a Sorvall Ultra80 ultracentrifuge.

Viral infectivity was measured by infecting TZM-bl cells, a HeLa cell-derived cell line integrated with HIV-1 LTR promoter. Briefly, TZM-bl cells were seeded in a 24-well cell culture plate at a concentration of 4 × 10^4^ per well. Twenty-four hours later, TZM-bl cells were infected with resulting virus, which has been normalized by the amount of p24, in the presence of 20 μg/ml DEAE dextran (Catalog No. 00898, Sigma). At 48 h after infection, the TZM-bl cells were harvested and lysed, and luciferase activity was measured using the Promega Dual-Luciferase Reporter Assay System (Catalog No. E2810; Promega) according to the manufacturer’s protocol with a GloMax 20/20 Luminometer (Promega).

### Chemical Synthesis of siRNA

To knock down endogenous CUL2 and CUL5, short interfering RNA (siRNA) against CUL2 and CUL5 and non-specific control were purchased from RiboBio (Guangzhou, China). The sequences of siRNAs have been described previously (Zhang W. et al., 2014; Su et al., 2018).

### Lentiviral Production and Transduction

To generate a stable cell line for CYPA knockdown, the shRNA against CYPA was subcloned into the pLKO.1-puro shRNA vector. Replication-defective vesicular stomatitis virus G protein pseudotyped lentiviruses were produced by transfecting HEK293T cells with shCYPA or pLKO.1 plus RRE, REV and VSV-G vectors using Lipofectamine 2000 (Invitrogen). At 48 h post transfection, the control lentivirus or the lentivirus containing shRNA targeting CYPA was collected to infect HEK293T cells. Forty-eight hours after infection, puromycin (1 μg/ml) was added to the culture medium for selection. Five to 7 days later, expression of CYPA was examined by immunoblotting analysis, and subsequent experiments were performed.

### Immunoblot Analysis

HEK293T cells were harvested at 48 h after transfection and lysed in 1 ( × loading buffer (0.08M Tris, pH 6.8, with 2.0% SDS, 10% glycerol, 0.1M dithiothreitol and 0.2% bromophenol blue). The samples were boiled for 30 min and the proteins were separated by 12% SDS-polyacrylamide gel electrophoresis (PAGE) and transferred onto polyvinylidene fluoride membranes, which was then incubated with primary antibodies against the indicated proteins and subsequently HRP-conjugated secondary antibodies. Next, the membranes were washed in TBST three times and incubated with chemiluminescent HRP substrate (Millipore, Burlington, MA, United States) according to the manufacturer’s instruction. The bands were visualized by multi-functional number imaging system (Azure, CYCLOUD, Beijing, CHN).

The anti-HA antibody (Catalog No. MMS-101R-10000, Covance), anti-actin antibody (catalog no. A00702-100, Genscript, Piscataway, NJ, United States), anti-p24 antibody (Catalog No. 1513, AIDS Research and Reference Reagents Program,) anti-Cul5 antibody (Catalog No. sc-13014, Santa Cruz Biotechnology, United States), anti-Cul2 antibody (Catalog No. sc-10781, Santa Cruz Biotechnology), anti-ELOB antibody (Catalog No. sc-10781, Santa Cruz Biotechnology), anti-CBF-β antibody (Catalog No. sc-166142, Santa Cruz Biotechnology), anti-CYPA antibody (Catalog No. D222908, BBI life Sciences) were used as the primary antibodies. HRP-conjugated Goat anti-Rabbit IgG (Catalog No. D110058; BBI life Sciences) and HRP-Goat anti-Mouse IgG (catalog no. NC-AP124P-200; millipore) were used as the secondary antibodies.

### Immunoprecipitation

To determine the interaction between CAEV Vif wild type (WT) or its mutants and cellular partners, HEK293T cells were transfected with HA-tagged expression vectors of WT or mutants of CAEV Vif. At 48 h post transfection, the HEK293T cells were harvested and washed twice with cold PBS, followed by dissociating in lysis buffer (PBS containing 1% Triton X-100 and complete protease inhibitor cocktail tablets [Roche, Mannheim, Germany]) at 4 °C for 30 min. The pre-cleared supernatants were collected by centrifugation at 10,000 g for 30 min at 4 °C and then mixed with anti-HA antibody conjugated agarose beads (cCatalog nNo. 190–119, Roche, Mannheim, Germany) at 4 °C for 3 h or overnight on an end-over-end rocker. Subsequently, the mixtures were washed six times with cold washing buffer (20 mM Tris, pH 7.5, 100 mM NaCl, 0.1 mM EDTA and 0.05% Tween 20). The binding proteins were then resuspended in elution buffer (0.1 M glycine-HCl, pH 2.0) and detected by immunoblotting with indicated antibodies.

### Data Analysis

All data represent three repetitions of each experiment and are presented as means ± standard deviations (SDs). Statistical significance was analyzed by using Student’s *t*-test. Significant differences are indicated as follows: ^∗^*P* < 0.05, and ^∗∗^*P* < 0.01.

## Results

### CAEV Vif Degrades oaA3Z2-Z3 and Neutralizes Its Antiviral Activity Through a Proteasomal Pathway

**FIGURE 1:**
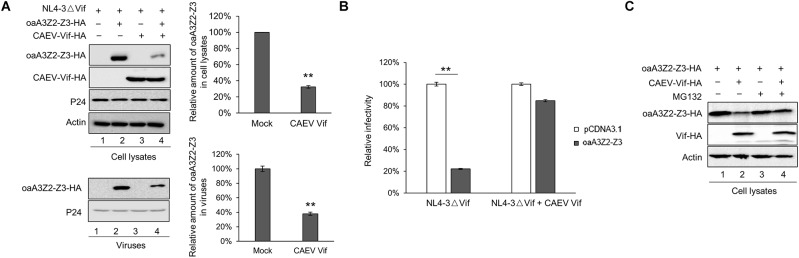
CAEV Vif degrades oaA3Z2-Z3 and impairs its antiviral activity through a proteasomal pathway. **(A)** CAEV Vif reduced oaA3Z2-Z3 expression and its incorporation into virions. HEK293T cells in 12-well plates were co-transfected with 500 ng of HIV-1 Vif-deficient molecular clone NL4-3ΔVif plus 300 ng of VR1012 or CAEV Vif in the absence or presence of 100 ng of oaA3Z2-Z3. Forty-eight hours later, cells and supernatant were harvested, and levels of indicated proteins were detected by immunoblotting and analyzed with the ImageJ software. **(B)** CAEV Vif impaired the antiviral activity of oaA3Z2-Z3. The infectivity of the resulting virus was assayed by infecting TZM-bl cells with supernatant for another 48 h and examining the luciferase activity. Virus infectivity in the absence of oaA3Z2-Z3 was set at 100%, and data are presented as means ± standard deviations (SDs) for triplicate wells. **(C)** MG132 inhibited CAEV Vif-induced oaA3Z2-Z3 degradation. HEK293T cells in 12-well plates were co-transfected with 100 ng of oaA3Z2-Z3 plus 300 ng of VR1012 or CAEV Vif. Thirty-six hours later, cells were treated with 10 μM MG132 or DMSO for another 12 h. Then the cells were harvested for immunoblotting with the indicated antibodies.

Previous studies have reported that oaA3Z2-Z3 protein is capable of restricting the infectivity of HIV-1 (Jonsson et al., 2006), and CAEV Vif can facilitate the degradation of oaA3Z2-Z3 (Ai et al., 2014; Yoshikawa et al., 2016). However, whether CAEV Vif could antagonize the antiviral activity of oaA3Z2-Z3 is still unclear. To explore this issue, we transfected HEK293T cells with the HIV-1 Vif-deficient molecular clone NL4-3ΔVif plus the empty vector VR1012 as a negative control or CAEV Vif in the absence or presence of oaA3Z2-Z3. As shown in Figure 1A, the CAEV Vif degraded oaA3Z2-Z3 and reduced its incorporation into HIV-1 NL4-3ΔVif virions (Figure 1A, lane 4) compared with the result in the absence of CAEV Vif (Figure 1A, lane 2). To further assess the viral infectivity, the supernatant from transfected cells was collected to infect TZM-bl cells. The infectivity of viruses produced by the HEK293T cells transfected with NL4-3ΔVif plus the control vector was set at 100%. In the absence of CAEV Vif, oaA3Z2-Z3 expression suppressed the infectivity of NL4-3ΔVif by ∼80% with statistical significance, but when co-transfected with CAEV Vif, the antiviral activity of oaA3Z2-Z3 was abolished (Figure 1B), implying that CAEV Vif could restore HIV-1 infectivity. Collectively, these results indicated that CAEV Vif decreased the incorporation of oaA3Z2-Z3 into the virus and impaired its antiviral activity.

Vif protein encoded by HIV-1 and some other lentiviruses have been demonstrated to degrade human and artiodactyl A3 proteins, respectively, in a proteasome-dependent manner (Yu et al., 2003; Zhang W. et al., 2014; Su et al., 2018). Thus, we investigated whether polyubiquitination and the proteasomal degradation pathway play a crucial role in the regulation of oaA3Z2-Z3 by CAEV Vif. HEK293T cells were transfected with oaA3Z2-Z3 in the absence or presence of CAEV Vif. Thirty-six hours after their transfection, the cells were treated with 10 μM MG132 proteasome inhibitor or with dimethyl sulfoxide (DMSO) as a negative control. Compared with the DMSO-treated cells (Figure 1C, lane 2), the degradation of oaA3Z2-Z3 by CAEV Vif was blocked in the presence of MG132 (Figure 1C, lane 4), suggesting that CAEV Vif overcomes the antiviral function of oaA3Z2-Z3 by degrading it via a proteasomal pathway.

### CAEV Vif Hijacks the Cellular Factors ELOB/C, CUL5 and CYPA, but Not CUL2 or CBF-β, as Partners to Form an E3 Ubiquitin Ligase Complex

**FIGURE 2:**
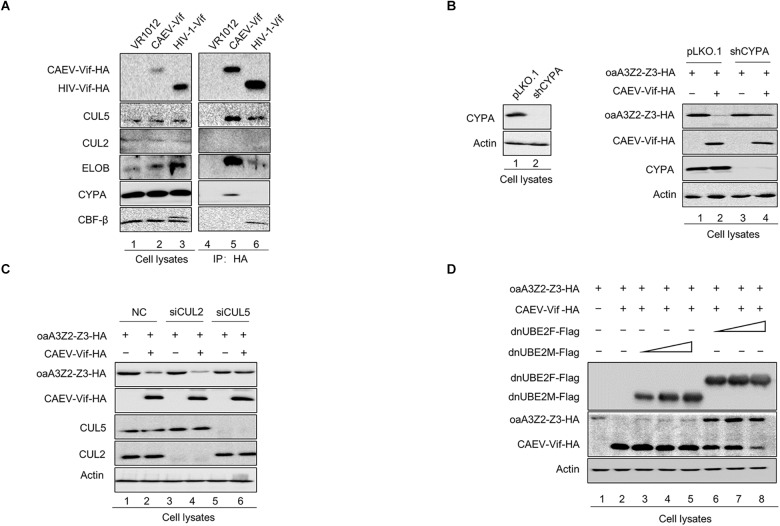
Interaction of CAEV Vif with cellular factors. **(A)** BIV Vif hijacked endogenous ELOB/C, CUL5 and CYPA, but not CUL2 or CBF-β. HEK293T cells in 6-cm dishes were transfected with 5 (μg of HIV Vif-HA or CAEV Vif-HA. Forty-eight hours later, cell lysates were immunoprecipitated with anti-HA antibody-conjugated agarose beads and examined by immunoblotting with antibodies against HA, CUL5, CUL2 ELOB, CBF-(β, and CYPA. **(B)** The silencing of CYPA inhibited the CAEV Vif-mediated degradation of oaA3Z2-Z3. To generate a stable cell line for CYPA knockdown, a control lentivirus or the lentivirus containing CYPA-specific shRNA was used to infect the HEK293T cells. Both the control and shCYPA HEK293T cells were co-transfected with 100 ng of osA3Z2-Z3 plus 300 ng of CPYA or control vector as indicated. At 48 h after transfection, the cells were harvested and detected by immunoblotting with the indicated antibodies. **(C)** Silencing CUL5, but not CUL2, suppressed the CAEV Vif-induced degradation of oaA3Z2-Z3. HEK293T cells were transfected with oaA3Z2-Z3 plus an siRNA pool targeting CUL2 or CUL5 or non-targeting control siRNA in the absence or presence of CAEV Vif. The levels of indicated proteins were analyzed by immunoblotting. **(D)** CAEV Vif used the RBX2-UBE2F neddylation system to degrade oaA3Z2-Z3. HEK293T cells in 12-well plates were transfected with 100 ng of oaA3Z2-Z3 and 300 ng of VR1012 or CAEV Vif plus increasing amounts (100, 300, and 900 ng, respectively) of UBE2F (C116S) or UBE2M (C111S). Forty-eight hours later, the cells were harvested and detected by immunoblotting with the indicated antibodies.

The involvement of host cellular factors is required for the Vif-induced degradation of A3 proteins, and the cellular factors recruited by different lentiviral Vif proteins are virus-specific (Zhang et al., 2018). For instance, HIV-1 Vif hijacks CUL5, ELOB/C and CBF-β to neutralize the antiviral activity of human A3 proteins (Jager et al., 2011; Zhang et al., 2011), while Cullin 2 (CUL2), ELOB/C, and RBX1, but not CUL5 or CBF-β, were employed by BIV and JDV Vif to form a CRL2 E3 ubiquitin ligase to degrade the restrictive bovine A3 proteins (Zhang W. et al., 2014; Su et al., 2018). To identify the cellular factors recruited by CAEV Vif for degrading oaA3Z2-Z3 to a comprehensive degree, we transfected HEK293T cells with CAEV Vif -HA, VR1012 as a negative control or HIV-1 Vif-HA as a positive control and performed a co-immunoprecipitation assay. By using the anti-HA antibody-conjugated agarose beads to immunoprecipitate HA-tagged Vif, we observed that HIV Vif interacted with endogenous CUL5, ELOB/C and CBF-β instead of other host factors (Figure 2A, lane 6), reflecting the efficiency and specificity of the assay. CAEV Vif was found to bind with CUL5 and ELOB/C, but not CUL2 (Figure 2A, lane 5), which is consistent with HIV-1 Vif. Nevertheless, CBF-β, the non-canonical cofactor required by primate lentivirus Vif (Yoshikawa et al., 2015), failed to co-precipitate with CAEV Vif. By contrast, cellular protein cyclophilin A (CYPA), which is a partner of the small ruminant lentiviruses (Kane et al., 2015; Yoshikawa et al., 2016), was capable of interacting with CAEV Vif (Figure 2A, lane 5).

Subsequently, to further confirm the participation of host factors in the CAEV Vif-mediated degradation of ovine APOBEC3, we employed a loss of function approach. The results showed that when CYPA was stably knocked down in HEK293T cells (Figure 2B, left panel), the degradation of oaA3Z2-Z3 induced by CAEV Vif was significantly impaired (Figure 2B, lane 4 of right panel) compared with that of the control HEK293T cells (Figure 2B, lane 2 of right panel). Moreover, silencing the endogenous CUL5 with specific siRNA (Figure 2C, lane 6) impaired the CAEV Vif-mediated degradation of oaA3Z2-Z3 (Figure 2C, lane 2), but silencing CUL2 did not (Figure 2C, lane 4).

RBX1-UBE2M and RBX2-UBE2F are two well-known pathways for cullin neddylation (Wada et al., 2000), which is required for the activation of Cullin-RING E3 ubiquitin ligases (CRLs) (Deshaies et al., 2010). We then examined which pathway was used in the CAEV Vif-mediated degradation of oaA3Z2-Z3. Dominant negative mutants of UBE2M (C111S) or UBE2F (C116S) were used in this assay. The UBE2F dominant negative mutant (C116S) was found to markedly diminish the CAEV Vif-mediated oaA3Z2-Z3 degradation in a dose-dependent manner (Figure 2D, lanes 6–8), in stark contrast with the UBE2M dominant negative mutant (C111S), which did not affect the CAEV Vif function (Figure 2D, lanes 3–5), indicating that CAEV Vif uses the RBX2-UBE2F neddylation system to degrade oaA3Z2-Z3.

Taken together, these results demonstrated that CUL5, ELOB/C CYPA, and RBX2 were essential for CAEV to degrade oaA3Z2-Z3 by forming a CRL5 E3 ubiquitin ligase complex.

### Identification of SLE Motif of CAEV Vif as the BC Box

**FIGURE 3:**
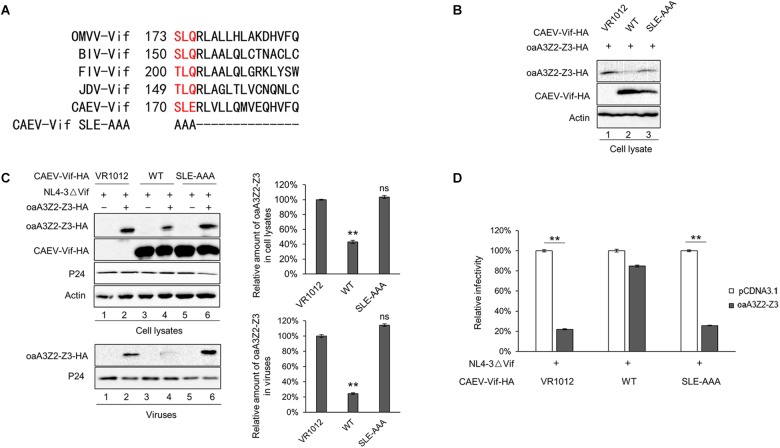
Identification of the SLE motif in CAEV Vif as the BC box. **(A)** Alignment of the BC box sequence of various Vif proteins as well as the CAEV Vif SLE-deficient mutation constructed in this study. **(B)** The BC box in CAEV Vif is required for the CAEV Vif-mediated degradation of oaA3Z2-Z3. HEK293T cells were co-transfected with oaA3Z2-Z3 plus the WT or the SLQ-AAA mutant of CAEV Vif. The cells were harvested and detected by immunoblotting analysis. **(C)** The SLQ-AAA mutant of CAEV Vif was deficient in preventing the packaging of oaA3Z2-Z3 into virions. NL4-3ΔVif with the WT or the SLQ-AAA mutant of CAEV Vif in the absence or presence of oaA3Z2-Z3 was co-transfected into HEK293T cells. Forty-eight hours later, the cells and supernatant were harvested and detected by immunoblotting and analyzed using ImageJ software. **(D)** CAEV Vif impaired the antiviral activity of oaA3Z2-Z3. The infectivity of the producing virus was detected as described in Figure 1B, and the virus infectivity in the absence of oaA3Z2-Z3 was set at 100%. The data are presented as the means ± SDs for triplicate wells. Statistical significance was analyzed by using Student’s *t*-test. Significant differences are indicated as follows: ^∗^*P* < 0.05 and ^∗∗^*P* < 0.01.

It has been reported that an interaction with ELOB/C through the BC box of Vif is a prerequisite for assembling a substrate receptor to bind CUL2 or CUL5 (Guo et al., 2014; Zhang W. et al., 2014; Gu et al., 2018; Su et al., 2018). To characterize the functional domains of CAEV Vif, we aligned the sequences of CAEV Vif with other adaptor proteins. As shown in Figure 3A, an SLE motif of CAEV Vif was similar to the SLQ motif of OMVV and BIV Vif as well as the TLQ motif of FIV and JDV Vif, which have been found to act as BC boxes (Wang et al., 2011; Zhang J. et al., 2014; Zhang W. et al., 2014; Su et al., 2018). Therefore, to examine whether the BC box was essential for the degradation of oaA3Z2-Z3 as induced by CAEV Vif, we replaced the SLE sequence of CAEV Vif with AAA. The degradation assay revealed that the CAEV Vif SLE-AAA mutant failed to degrade oaA3Z2-Z3 (Figure 3B, lane 3) compared with the WT (Figure 3B, lane 2). We then further investigated the effect of the CAEV Vif SLE-AAA mutant on the antiviral activity of oaA3Z2-Z3. As expected, the SLE-AAA mutant of CAEV Vif lost the ability to block the packaging of oaA3Z2-Z3 into virions (Figure 3C) and was also deficient in inhibiting the antiviral activity of oaA3Z2-Z3 (Figure 3D). Thus, these results implied that the SLE motif of CAEV Vif is required for its degradation of oaA3Z2-Z3.

### CAEV Vif P21 Is Required for Its Function

**FIGURE 4:**
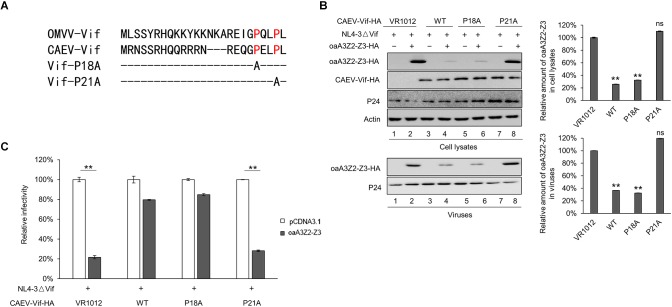
CAEV Vif P21 is required for its function. **(A)** Sequence alignment of various Vif proteins referenced to the speculated CYPA binding region and the mutations constructed in this study. **(B)** Mutated P21 but not P18 of the CAEV Vif mutant lost the ability to degrade and inhibit the incorporation of oaA3Z2-Z3 into virions. NL4-3ΔVif with WT, P18A or P21A mutants of CAEV Vif in the absence or presence of oaA3Z2-Z3 was co-transfected into HEK293T cells. Forty-eight hours later, the cells and supernatant were harvested and detected by immunoblotting assay and analyzed with ImageJ software. **(C)** The P21A mutant of CAEV Vif could not rescue NL4-3ΔVif infectivity in the presence of oaA3Z2-Z3. The infectivity of the virus produced in **(C)** was detected as described in Figure 1B, and the virus infectivity in the absence of oaA3Z2-Z3 was set at 100%. The data are presented as the means ± SDs for triplicate wells. Statistical significance was analyzed by using Student’s *t*-test. Significant differences are indicated as follows: ^∗^*P* < 0.05 and ^∗∗^*P* < 0.01.

As a member of the MVV Vif-induced CRL5 complex, CYPA has been reported to bind with residues P21 and P24 of the MVV Vif (Kane et al., 2015). To identify the corresponding region of the CAEV Vif for CYPA binding, we aligned the sequence of CAEV Vif with that of MVV Vif and found that residues P18 and P21 in the N-terminus of CAEV Vif were putative binding sites (Figure 4A). We then mutated either of these two sites into residue A and examined the effect on the CAEV Vif function. As shown in Figure 4B, compared with the WT CAEV Vif (lane 4), the P18A mutant could still degrade oaA3Z2-Z3 (lane 6), which was not observed in P21A (lane 8), implying that residue P21 was critical for the degradation of oaA3Z2-Z3 induced by CAEV Vif. Moreover, CAEV Vif P21A also lost the ability to prevent the incorporation of oaA3Z2-Z3 into virions (Figure 4B, lower panel) or suppress the antiviral activity of oaA3Z2-Z3 (Figure 4C). Collectively, these findings indicated that residue P21 was crucial for CAEV Vif function.

### C132-C134-C154-C157 Motif Is Required for CAEV Vif to Induce the Degradation of the oaA3Z2-Z3 Protein

**FIGURE 5:**
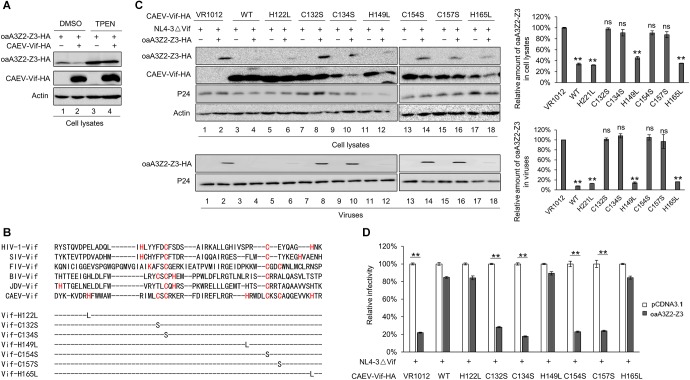
C132-C134-C154-C157 motif is required for CAEV Vif to induce the degradation of osA3Z2-Z3 protein. **(A)** TPEN inhibited CAEV Vif-induced oaA3Z2-Z3 degradation. HEK293T cells were co-transfected with 100 ng of oaA3Z2-Z3 plus 300 ng of VR1012 or CAEV Vif expression vectors. Twenty-four hours later, the cells were treated with 2.5 μM TPEN or DMSO for another 24 h. The cells were then harvested and analyzed by immunoblotting with indicated antibodies. **(B)** Multiple sequence alignments of various Vif proteins and CAEV Vif mutants with mutations referenced to the zinc binding motif constructed in this study. **(C)** C132-C134-C154-C157 mutations were deficient in counteracting the antiviral activity of oaA3Z2-Z3. HEK293T cells were co-transfected with NL4-3ΔVif plus CAEV Vif WT or each mutant of the predicted zinc binding motif in the absence or presence of oaA3Z2-Z3. Forty-eight hours later, the cells and supernatant were collected. The levels of the indicated proteins were detected by immunoblotting assay and analyzed with ImageJ software. **(D)** The infectivity of the virus produced in **(C)** was measured by detecting the luciferase activity after infecting the TZM-bl cells with supernatant for another 48 h. The infectivity of cells transfected with NL4-3ΔVif plus control vector was set at 100%. The data are presented as the means ± SDs for triplicate wells. Statistical significance was analyzed by using Student’s *t*-test. Significant differences are indicated as follows: ^∗^*P* < 0.05 and ^∗∗^*P* < 0.01.

The zinc finger motif in lentivirus Vif proteins is indispensable for the Vif-induced degradation of A3 proteins (Zhang W. et al., 2014; Gu et al., 2018; Su et al., 2018). To test whether this was also the case for CAEV Vif, we examined the effect of TPEN, a membrane-permeable zinc chelator, on the function of CAEV Vif. Expression plasmids of oaA3Z2-Z3 plus VR1012 or CAEV Vif-HA were transfected into HEK293T cells. Twenty-four hours later, the cells were treated with 2.5 μM TPEN or DMSO as a control for another 24 h. In the DMSO-treated cells, CAEV Vif degraded oaA3Z2-Z3 as expected (Figure 5A, lane 2), but this effect was impaired in the presence of 2.5 μM TPEN (Figure 5A, lane 4), indicating that the zinc finger motif in CAEV Vif may be critical for its function. To identify the zinc finger motif in CAEV Vif, we aligned its sequence with those of other Vif proteins, and we found several histidines and cysteines (Figure 5B). We then constructed corresponding mutants of each site (H122L, C132S, C134S, H149L, C154S, C157S, and H165L). As shown in Figure 5C, the H122L, H149L, and H165L mutants did not affect the CAEV Vif-induced degradation of oaA3Z2-Z3 (lanes 6, 12, and 18), but the C132S, C134S, C154S, and C157S mutations lost the ability to suppress oaA3Z2-Z3 (lanes 8, 10, 14, and 16). The mutants C132S, C134S, C154S, and C157S were also deficient in preventing the packaging of oaA3Z2-Z3 into virions (Figure 5C, lower panel) and counteracting the antiviral activity of oaA3Z2-Z3 (Figure 5D). These results suggest that the C132-C134-C154-C157 motif in CAEV Vif is critical for the function of the protein by acting as a zinc finger motif.

### 141IR142 in the Hydrophobic Domain Is Essential for CAEV Vif Function

**FIGURE 6:**
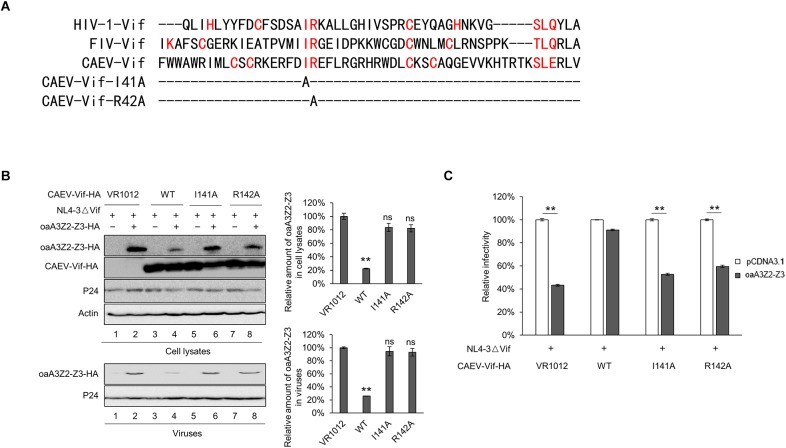
141IR142 in the hydrophobic domain is essential for CAEV Vif function. **(A)** Sequence alignment of various Vif proteins referenced to the hydrophobic domain and the mutations constructed in this study. **(B)** I141A and R142A of CAEV Vif was deficient in degrading oaA3Z2-Z3 or preventing the packaging of oaA3Z2-Z3 into virions. NL4-3ΔVif with WT, I141A or R142A mutants of CAEV Vif were co-transfected into HEK293T cells in the absence or presence of oaA3Z2-Z3. Forty-eight hours later, the cells and viruses were detected by immunoblotting and analyzed with ImageJ software. **(C)** I141A and R142A of CAEV Vif could not restore the NL4-3ΔVif infectivity. The infectivity of the virus produced in **(B)** was measured as described in Figure 1B, and the virus infectivity in the absence of oaA3Z2-Z3 was set at 100%. The data are presented as the means ± SDs for triplicate wells. Statistical significance was analyzed by using Student’s *t*-test. Significant differences are indicated as follows: ^∗^*P* < 0.05 and ^∗∗^*P* < 0.01.

We further identified the CUL5 binding sites of CAEV Vif. As recently reported, conserved hydrophobic domains localized in the zinc-binding motif of HIV-1 and FIV were essential for their direct interaction with CUL5 (Gu et al., 2018). To investigate whether there was a corresponding hydrophobic domain (IR) in the CAEV Vif involved in CUL5 binding, we performed a sequence alignment and replaced the 141IR142 with alanines (Figure 6A). The results showed that both I141A and R142A lost the ability to degrade oaA3Z2-Z3 (Figure 6B, upper panel), and they were also deficient in preventing the incorporation of oaA3Z2-Z3 into virions (Figure 6B, lower panel) or suppressing the antiviral activity of oaA3Z2-Z3 (Figure 6C). From these experiments, we conclude that the 141IR142 in the hydrophobic domain is essential for CAEV Vif function.

### E3 Ubiquitin Ligase Complex Formed by CAEV Vif Is Assembled Stepwise

**FIGURE 7:**
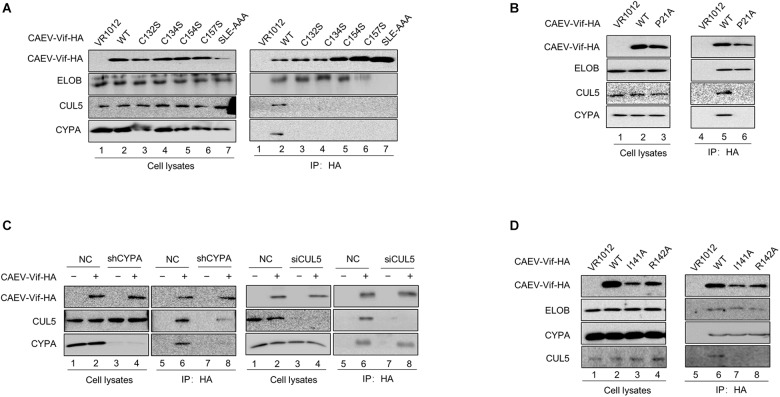
CAEV Vif assembled E3 ubiquitin ligase complex in a stepwise fashion. **(A)** Binding with ELO/BC on the BC box was the initial step for CAEV Vif to form E3 ubiquitin ligase. HA-tagged expression vector of WT or mutants of CAEV Vif were used to transfect HEK293T cells. Forty-eight hours after the transfection, the cells were harvested and an immunoprecipitation assay was performed. The levels of the indicated proteins were measured by immunoblotting with antibodies against HA, ELOB, CUL5 and CYPA. **(B)** P21A mutant of CAEV Vif failed to bind with either CYPA or CUL5. HEK293T cells in 6-cm dishes were transfected with 5 μg of WT or the P21 mutant of CAEV Vif-HA. Forty-eight hours later, the cell lysates were immunoprecipitated with anti-HA antibody-conjugated agarose beads and detected by immunoblotting with antibodies against HA, CUL5, ELOB, and CYPA. **(C)** Silencing of endogenous CYPA impaired binding with CUL5 of CAEV Vif, whereas the silencing of endogenous CYPA has no effect on binding with the CYPA of CAEV Vif. (Left panel) Control or CYPA stably knocked down the HEK293T cells described in Figure 2, which were seeded in 6-cm dishes and transfected with 5 μg of the empty vector VR1012 or HA-tagged CAEV Vif. The cell lysates were immunoprecipitated with anti-HA antibody-conjugated agarose beads and analyzed by immunoblotting assay. (Right panel) HEK293T cells were transfected with HA-tagged CAEV Vif plus the siRNA pool targeting CUL5 or the non-targeting control. The levels of the indicated proteins were analyzed by immunoblotting. Forty-eight hours later, CAEV Vif-binding protein were pulled down with anti-HA antibody-conjugated agarose beads and analyzed by immunoblotting assay. **(D)** I141A and R142A of CAEV Vif failed to bind with CUL5. HEK293T cells in 6-cm dishes were transfected with 5 μg of WT, I141A or R142A mutants of CAEV Vif-HA. Forty-eight hours later, the cell lysates were immunoprecipitated with anti-HA antibody-conjugated agarose beads and detected by immunoblotting with antibodies against HA, CUL5, ELOB, and CYPA.

Having observed the interaction between the functional regions of CAEV Vif with their partners, we then performed a series of co-immunoprecipitation experiments to clarify the assembly process of CAEV Vif-mediated E3 ligase. HEK293T cells were transfected with the HA-tagged expression vector of WT or mutant CAEV Vif, and VR1012 was used as an empty control. The results revealed that when the SLE motif of the BC box was defective, CAEV Vif could no longer bind with EloB/C, as well as CUL5 and CYPA, which validated the initial role of the BC box involved assembling a substrate receptor for CAEV Vif function (Figure 7A, lane 7). In addition, when the residues in the zinc finger motif were mutated (C132S, C134S, C154S, and C157S), the interaction between CAEV Vif and CUL5 was abolished, as expected (Figure 7A, lanes 3–6). Besides, C132S, C134S, C154S, and C157S also lost the ability to bind CYPA (Figure 7A, lanes 3–6). Notably, when the CPYA binding site P21A was mutated, CAEV Vif was also deficient in binding with CUL5 (Figure 7B). Next, to explore the underlying mechanism of the interplay among CAEV Vif, CUL5 and CYPA, we silenced endogenous CYPA with shRNA, and we found that when CYPA was knocked down, the association of CAEV Vif with CUL5 decreased (Figure 7C, lane 8 of left panel), implying that the interaction of CAEV Vif with CUL5 was mediated by CYPA. By contrast, when endogenous CUL5 was silenced by siRNA, the binding of CAEV Vif and CYPA was not affected (Figure 7C, lane 8 of right panel). These results are consistent with the finding observed in Figure 7B, in which the association with CYPA was a prerequisite for CUL5 binding. In addition, we further examined whether the CAEV Vif-CYPA association would be affected when the CUL5 binding sites was defective. As shown in Figure 7D, compared with the WT (lane 6), both the I141A and R142A mutants of CAEV Vif were not capable of binding CUL5 (lanes 7–8), which confirmed that the hydrophobic domain (141IR142) of CAEV Vif was indispensable for CUL5 binding. The interaction of CAEV Vif and CYPA was not affected, indicating that the interaction with CUL5 is likely more of a downstream step than CYPA binding. From these experiments, we conclude that the E3 ubiquitin ligase complex formed by CAEV Vif is assembled stepwise. In brief, CAEV Vif first binds ELOB/C to form a substrate receptor; then, CAEV Vif-ELOB/C interacts with cellular factor CYPA, which may play a similar role to that of CBFβ-like in regulating ligase assembly; finally, CAEV Vif-ELOB/C-CYPA recruits CUL5 to form a complete E3 ligase to degrade oaA3Z2-Z3.

### N-Terminal Residues Y39 and L44 of CAEV Vif Contribute to oaA3Z2-Z3 Binding

**FIGURE 8:**
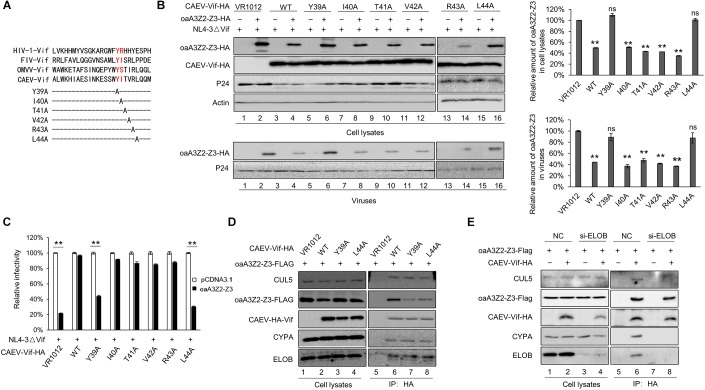
N-terminal residues Y39 and L44 of CAEV Vif are indispensable for binding oaA3-Z2-Z3. **(A)** N-terminal sequence of CAEV Vif was aligned with other Vif proteins. **(B)** Y39A and L44A mutations of CAEC Vif failed to induce oaA3Z2-Z3 degradation and were deficient in antagonizing the antiviral activity of oaA3Z2-Z3. HEK293T cells were co-transfected with NL4-3ΔVif plus CAEV Vif WT or mutants of the predicted oaA3Z2-Z3 binding region in the absence or presence of oaA3Z2-Z3. Forty-eight hours after transfection, the cells and supernatant were collected and detected by immunoblotting and analyzed using ImageJ software. **(C)** The infectivity of the virus produced in **(B)** was detected as described in Figure 1B, and the infectivity of the cells transfected with NL4-3ΔVif plus the control vector was set as 100%. The data are presented as the means ± SDs for triplicate wells. **(D)** Y39A and L44A mutations of CAEV Vif lost the ability to bind oaA3Z2-Z3. HEK293T cells were transfected with Flag-tagged oaA3Z2-Z3 and HA-tagged WT or mutant CAEV as indicated. At 36 h post-transfection, the HEK293T cells were treated with 10 μM MG132 proteasome inhibitor for another 12 h. Then, the binding between the WT or mutant CAEV Vif and oaA3Z2-Z3 was detected by pulling down the interaction complex with anti-HA antibody-conjugated agarose beads and measuring with immunoblotting assay. **(E)** CAEV Vif interacts with oaA3Z2-Z3 directly. HEK293T cells were transfected with oaA3Z2-Z3 plus the siRNA pool targeting ELOB or the non-targeting control siRNA in the absence or presence of CAEV Vif. At 36 h post-transfection, the HEK293T cells were treated with 10 μM MG132 proteasome inhibitor for another 12 h. Then, the HEK293T cells were harvested and an immunoprecipitation assay was performed. The levels of the indicated proteins were analyzed by immunoblotting. Statistical significance was analyzed by using Student’s *t*-test. Significant differences are indicated as follows: ^∗^*P* < 0.05 and ^∗∗^*P* < 0.01.

By aligning the N-terminal sequence of CAEV Vif with FIV Vif, domain 39–44 was identified as the putative domain for oaA3Z2-Z3 binding (Figure 8A). We then performed site-directed mutagenesis that spanned this region to identify amino acid residues that are critical for the Vif neutralization of oaA3Z2-Z3. The results revealed that the degradation of oaA3Z2-Z3 was not affected when residues I40, T41, V42 and R43 of CAEV Vif were replaced (Figure 8B, lanes 8, 10, 12, and 14); however, Y39A and L44A both lost the ability to degrade oaA3Z2-Z3 (Figure 8B lanes 6 and 16) compared with WT CAEV Vif (Figure 8B, lane 4). Moreover, Y39A and L44A were also unable to prevent the packaging of oaA3Z2-Z3 into virions (Figure 8B, lanes 6 and 16 of the lower panel) or restoring the HIV-1 infectivity (Figure 8C), implying the role of Y39 and L44 in CAEV Vif function. To further test whether these two sites directly affect the oaA3Z2-Z3 binding of CAEV Vif, we performed an immunoprecipitation assay. After anti-HA beads were used, WT CAEV Vif was found to bind oaA3Z2-Z3 (Figure 8D, lane 6), while the interaction between oaA3Z2-Z3 and Y39A (Figure 8D, lane 7) or L44A (Figure 8D, lane 8) was reduced significantly. Based on these results, we conclude that residues Y39 and L44 of CAEV Vif contribute to its oaA3Z2-Z3 binding. In addition, we further examine whether CAEV Vif-oaA3 binding involved the E3 ubiquitin ligase complex or CAEV Vif itself. As shown in Figure 8E, when endogenous ELOB was knocked down, CAEV Vif lost the ability to hijack CYPA or CUL5, which supported the initial role of CAEV Vif-EloB/C binding in assembling the E3 ligase complex as well. By contrast, the CAEV Vif-oaA3 binding was not affected by the absence of the CAEV Vif-mediated E3 ligase complex, indicating that CAEV-Vif could interact with aoA3 directly.

## Discussion

**FIGURE 9:**
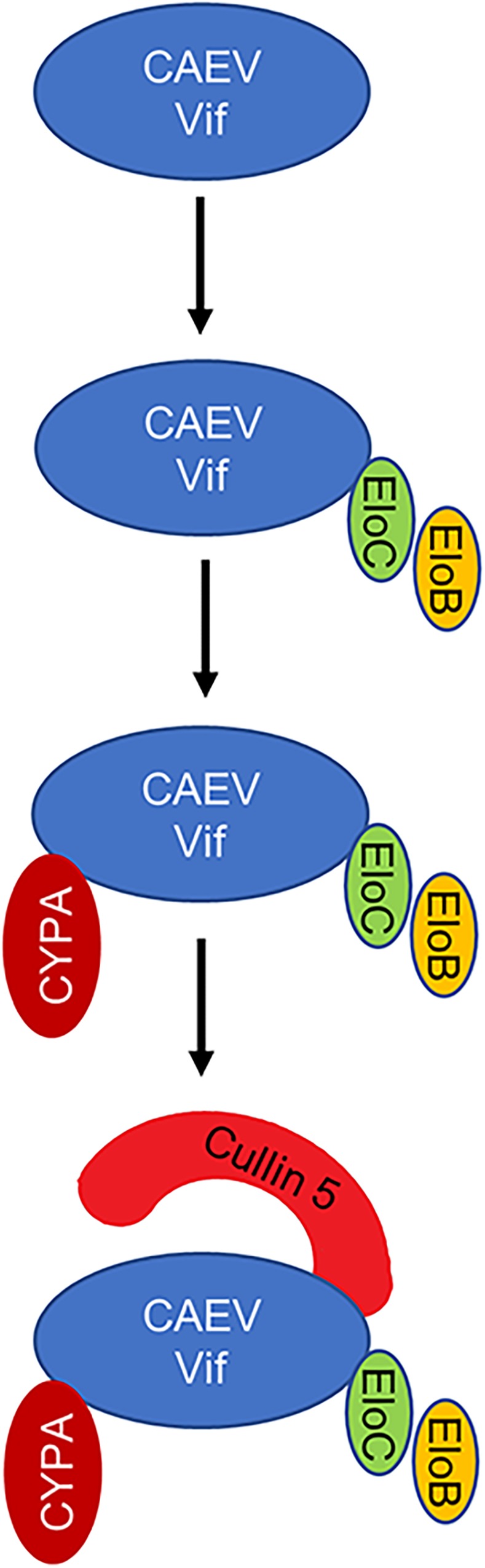
Schematic representation of assembly process of CAEV Vif-mediated E3 ligase. CAEV Vif initially binds ELOB/C at its SLE motif in the BC box (170SLE172) to form a substrate receptor; then, CAEV Vif-ELOB/C interacts with cellular factor CYPA at P21 of the 18PELP21 motif as well as the zinc finger motif (C132-C134-C154-C157); finally, CAEV Vif-ELOB/C-CYPA recruits CUL5 on the hydrophobic domain (141IR142) to form a complete E3 ligase to degrade oaA3Z2-Z3.

Caprine arthritis encephalitis virus is the pathogen that causes caprine arthritis-encephalitis, and it causes a slow, progressive and inflammatory pathology in multiple tissues and systems of sheep and goats. Like other lentiviruses, CAEV encodes Vif protein to escape from the antiviral restriction of OaA3Z2-Z3 as well (Ai et al., 2014). However, further detail about the interplay between CAEV Vif and OaA3Z2-Z3 is not clear. In this study, we identified the comprehensive cellular partner and functional domain of CAEV Vif as well as the assembly process of CAEV Vif-mediated E3 ligase for the first time. The results demonstrated that CAEV Vif initially binds ELOB/C at its SLE motif in the BC box (170SLE172); then, CAEV Vif-ELOB/C interacts with cellular factor CYPA at P21 of the 18PELP21 motif as well as the zinc finger motif (C132-C134-C154-C157). Subsequently, CAEV Vif-ELOB/C-CYPA recruits CUL5 on the hydrophobic domain (141IR142) to form a complete E3 ligase to degrade oaA3Z2-Z3 (Figure 9). Moreover, we identified the residues Y39 and L43 in CAEV Vif are required for oaA3Z2-Z3 direct interaction.

The lentivirus Vif proteins are functionally conserved to counteract the antiviral factor A3 proteins, and CAEV Vif is no exception. CAEV Vif neutralized the inhibition of oaA3Z2-Z3 and restored the infectivity of HIV-1, and the proteasome inhibitor MG132 significantly blocked this effect as expected (Figure 1). Regarding the precise detail of the CAEV Vif function, the cellular requirement and functional domain of CAEV Vif were identified in the present study. Unlike primate lentiviruses, including HIV-1 and SIV, which require the non-canonical cofactor CBF-(β as a key regulator in E3 ligase complex formation (Hultquist et al., 2012), CAEV Vif was found to bind CYPA instead, which was consistent with previous studies (Kane et al., 2015; Yoshikawa et al., 2016). This phenomenon was further supported by our finding that the stable knockdown of CYPA and loss of CYPA binding markedly suppressed the CAEV Vif counteraction against oaA3Z2-Z3 antiviral action (Figure 2B, 4B). As mentioned previously, CYPA has been identified as a cofactor for MVV, which was another member of SRLV with CAEV (Kane et al., 2015). When considering that HIV/SIV requires CBF-β and that BIV/JDV Vif requires no cofactor to counteract the antiviral activity of A3s, these findings strongly indicated that there is a preference in the cellular partners required by Vifs for different subgroups of lentiviruses (Nakano et al., 2017). This conservation and plasticity among diverse lentiviruses illustrate the co-evolutionary dynamics between viruses and hosts, and they provide informational clues for broad-spectrum pharmaceutical design.

It is notable that when CYPA binding site P21 was defective or when the endogenous CYPA was silenced, the CAEV Vif-CUL5 interaction was surprisingly diminished (Figure 7B,C), indicating that the CAEV Vif-CYPA interaction may affect CUL5 binding. In addition, the zinc finger motif (C132-C134-C154-C157), which was initially considered as a CUL5 binding site, showed CYPA binding potential (Figure 7A). Since RNAi against CUL5 had no effect on the association of CAEV Vif and CYPA (Figure 7C), the interaction of CAEV Vif with CYPA was likely more of an upstream step than CUL5 binding, and the zinc finger motif (C132-C134-C154-C157) was not the direct binding site for CUL5. This finding coincided with the observations that the zinc finger domain of HIV-1 contributed to Vif-CBF-β interactions and facilitated CUL5 binding, but not the direct binding sites for CUL5 (Fribourgh et al., 2014). Instead, consistent with HIV-1 and FIV (Gu et al., 2018), the hydrophobic domain (141IR142) of CAEV Vif was characterized as indispensable for CUL5 binding (Figure 7D). Thus, we concluded that the E3 ligase formed by CAEV Vif was assembled stepwise and CYPA played a key role in providing a prerequisite for CUL5 binding. Notably, the analogous role of CYPA and CBF-β was also supported by their evolutionary and structural conservation (Yoshikawa et al., 2016). In fact, these findings significantly suggested that CYPA was functionally analogous with CBF-β and may serve an essential role in enabling CAEV Vif to form an E3 ligase complex. Whether CYPA exerts this function via assisting CAEV Vif in maintaining an appropriate conformation or through other mechanisms as well as whether CYPA serve a similar role in MVV-mediated degradation of A3 requires future investigation.

In this study, we identified CAEV Vif 39YITVRL44 as what could be the oaA3-Z2-Z3-binding region by aligning the sequence of CAEV Vif with the corresponding region of FIV Vif, which has been found to determine its interaction with feline A3Z2 (Gu et al., 2016), and we found that mutating Y39 and L44 led to significant deficiencies in neutralizing the antiviral activity of oaA3-Z2-Z3 by losing most of the oaA3-Z2-Z3-binding ability (Figure 8). It was worth noting that when Y39 or L44 was mutated, CAEV Vif could still bind oaA3Z2-Z3 to a lesser extent, implying that residues Y39 and L44 are not the only residues participating in oaA3-Z2-Z3 binding, and the other residues that contribute to the CAEV Vif-oaA3Z2-Z3 interaction need further investigation. It has been reported that residue E289 of human A3F and residue R15 of HIV-1 Vif display a strong interaction through a “dock”-like manner (Richards et al., 2015); whether this is also the case for CAEV Vif needs additional study, and a structural analysis may be illustrative.

Given that both are members of the SRLV lentivirus sub-group, CAEV is closely related to MVV, and some similarities and differences could be assumed concerning their counteraction on oaA3Z2-Z3. For instance, they both require ELOB/C, CYPA and CUL5 to form the E3 ligase complex for degrading oaA3Z2-Z3. Notably, CYPA serves as a cofactor for both CAEV and MVV and plays an analogous role to that of CBF-β for primate lentiviruses. As mentioned above, MVV Vif binds with CYPA through residues P21 and P24 (Kane et al., 2015), whereas only residue P21 of CAEV Vif contributes to its interaction with CYPA. In addition, by comprehensively interpreting the cellular partners and functional domains, the CAEV Vif-mediated E3 ligase was found to be assembled stepwise, and whether MVV degrades oaA3Z2-Z3 through similar or different actions requires exploration in future studies.

By identifying the cellular requirement and functional domain of CAEV Vif as well as by illustrating the assembly process of CAEV Vif induced-E3 ligase, we have been able to depict the CAEV Vif function comprehensively, which may advance our understanding of the evolutionary arms race between lentiviruses and their hosts.

## Author Contributions

WZ, ZL, and CH conceived and designed the experiments and analyzed the data. CH and WZ wrote the manuscript. ZZ, XS, and HW performed the experiments. XS contributed reagents, materials, and analysis tools.

## Conflict of Interest Statement

The authors declare that the research was conducted in the absence of any commercial or financial relationships that could be construed as a potential conflict of interest.
